# Maximum Efficient Power Performance Analysis and Multi-Objective Optimization of Two-Stage Thermoelectric Generators

**DOI:** 10.3390/e24101443

**Published:** 2022-10-10

**Authors:** Lei Tian, Lingen Chen, Yanlin Ge, Shuangshuang Shi

**Affiliations:** 1 Institute of Thermal Science and Power Engineering, Wuhan Institute of Technology, Wuhan 430205, China; 2 Hubei Provincial Engineering Technology Research Center of Green Chemical Equipment, Wuhan 430205, China; 3 School of Mechanical & Electrical Engineering, Wuhan Institute of Technology, Wuhan 430205, China

**Keywords:** efficient power, finite-time thermodynamics, multi-objective optimization, optimal distribution of heat exchangers area, optimal distribution of thermoelectric elements, two-stage thermoelectric generator

## Abstract

Two-stage thermoelectric generators have been widely used in the aerospace, military, industrial and daily life fields. Based on the established two-stage thermoelectric generator model, this paper further studies its performance. Applying the theory of finite-time thermodynamics, the efficient power expression of the two-stage thermoelectric generator is deduced firstly. The maximum efficient power is obtained secondly by optimizing the distribution of the heat exchanger area, distribution of thermoelectric elements and working current. Using the NSGA-II algorithm, multi-objective optimizations of the two-stage thermoelectric generator are performed thirdly by taking the dimensionless output power, thermal efficiency and dimensionless efficient power as objective functions, and taking the distribution of the heat exchanger area, distribution of thermoelectric elements and output current as optimization variables. The Pareto frontiers with the optimal solution set are obtained. The results show that when the total number of thermoelectric elements is increased from 40 to 100, the maximum efficient power is decreased from 0.308W to 0.2381W. When the total heat exchanger area is increased from $0.03m^{2}$ to $0.09m^{2}$, the maximum efficient power is increased from 0.0603W to 0.3777W. The deviation indexes are 0.1866, 0.1866 and 0.1815 with LINMAP, TOPSIS and Shannon entropy decision-making approaches, respectively, when multi-objective optimization is performed on three-objective optimization. The deviation indexes are 0.2140, 0.9429 and 0.1815 for three single-objective optimizations of maximum dimensionless output power, thermal efficiency and dimensionless efficient power, respectively.

## 1. Introduction

Thermoelectric (TE) devices have been widely used in the aerospace, military, industrial, agricultural, medical, commercial and daily life fields by utilizing the TE effect. The TE effect [1,2,3] includes the Seebeck effect, the Peltier effect, the Thomson effect, the Fourier effect and the Joule effect. The Seebeck effect means that in a loop composed of two different conductors, if the temperatures at the two joints are different, electromotive force and direct current will be generated in the loop. The Peltier effect is the inverse effect of the Seebeck effect, which means that when a direct current passes through a loop composed of two different conductors, heat absorption or heat release will occur at the node. The Thomson effect means that when a current flows through a conductor with uneven temperature, the conductor will absorb or release a certain amount of heat in addition to the irreversible Joule heat. The Fourier effect is the thermal conductivity effect. The Joule effect is the thermal effect of the current. TE generators have many advantages over conventional power generation devices, such as environmental protection, miniaturization and long service life. Thus, to make full use of the TE elements in a limited space and increase the performance of the TE generator device, the TE generator device often adopts a two-stage element configuration. The main issue in the study of two-stage thermoelectric generators (TTEGs) is how to distribute TE elements at two stages optimally to achieve the maximum output power (P) and other performance indicators.

There are two main analytical methods for the study of TTEGs, namely, the traditional non-equilibrium thermodynamics (NET) theory and the finite-time thermodynamics (FTT) theory. In traditional NET, many scholars have not considered the finite rate heat transfer (HT) between the heat reservoir and the TE module in the research of TTEGs. For example, Atouei et al. [4] studied the TTEG system model through experiments, and the influence of different TE materials on the P and thermal efficiency (η) of the TTEG was analyzed. Pei et al. [5] designed a two-stage TE module through the studied TE device materials, and thereby improved the P and η of the TTEG. Ma et al. [6] considered a TTEG to absorb the waste heat of concentrating photovoltaic cells to improve energy utilization, and formed a new hybrid system. They compared the power density and η of concentrator photovoltaic cells and the TTEG with and without the Thomson effect.

The theory of FTT [7,8,9,10,11,12,13,14,15,16,17,18,19,20,21] has been applied to the study of TTEGs, fully considering the irreversibility between the heat reservoir and TE module, see review articles [10,14]. The influences of each parameter on the P, η and temperature change of the end face of TTEGs are analyzed. A series of new conclusions have been obtained that differ from the traditional NET analysis results. The research results are more realistic and can more accurately reflect the performance of the actual TTEGs. Chen et al. [22] optimized the distribution of the heat exchangers (HEXs) area and the distribution of the TE elements of the TTEG for maximum P and η firstly. Hans et al. [23] optimized P, η, voltage and current for the TTEG in series and parallel on the circuit. They analyzed the current–voltage characteristics in both cases and obtained the optimal operating load range. Asaadi et al. [24] analyzed the effect of the heat source temperature of the two-stage annular TE generator on energy and exergy performances through a three-dimensional numerical simulation. Zhang et al. [25] analyzed the effects of the Seebeck coefficient, HEX area changes and HT coefficient on the P and η of the TTEG. 

Yan [26] studied the cycle performance by using a product of P and η of an endoreversible Carnot heat engine as an optimization objective. Yilmaz [27,28] named the product of P and η as efficient power ($E_{P}$). As an optimization objective, the $E_{P}$ has been used in the studies of other heat engine cycles. Some scholars took the $E_{P}$ as the optimization objective, and studied the optimal performance of the Brayton cycles [29,30], law dissipative cycle [31], rectangular cycle [32] and single-stage TE generator [33], etc. 

The above optimization only considers single-objective performance. However, in actual cycles and devices, multiple optimization objectives are often considered, but multiple objective functions cannot reach the maximum values at the same time. Multi-objective optimization (MOO) can better coordinate multiple objective functions. Based on the NET model, MOOs of TTEGs have been performed. Liu et al. [34] used the height of the TE element module and area ratio of An/Ap as optimization variables, and carried out MOO of the P and η of the TTEG. Sun et al. [35] used load resistance, the area ratio of An/Ap, TE module height and angle between the TE elements as optimization variables, and carried out MOO of the entropy generation rate and specific power of the TTEG. 

Based on the FTT model, MOOs of TTEGs have also been performed. Arora et al. [36] used the operating current, and hot and cold end temperatures as optimization variables, and carried out MOO of the P, η and ecological function of the TTEG in series and parallel. Based on the same model, Arora et al. [37] used the operating current, hot and cold end temperatures and numbers of the upper and lower TTEG as optimization variables to perform MOO of the P, η and entropy generation rate. Arora et al. [38] also carried out MOO of the two-stage TE heat pump. They used the operating current, hot and cold end temperatures and first-stage TE element number as optimization variables, and carried out MOO of the heating load, coefficient of performance and ecological function of two-stage series and parallel TE heat pump. Sun et al. [39] used the operating current, lower TE element module height and ratio of the HEX channel width to fin thickness as optimization variables, and carried out two-objective optimization of the exergy efficiency and irreversibility of two-stage series and parallel TE refrigerators. The MOO of NSGA-II is also widely used in the Brayton cycle [40], Stirling–Otto combined cycle [41], Organic Rankine cycle [42], Stirling cycle [43,44], etc.

This study analyzes the effects of the total number of TE elements and the total HEX area on the $E_{P}$, and optimize the output current, distribution of HEXs area and distribution of TE elements. MOO of dimensionless P($\bar{P}$), η and dimensionless $E_{P}$(${\bar{E}}_{P}$) is performed using the NSGA-II algorithm. The innovations of this paper include: (1) Comparing the η of the TTEG at maximum P and $E_{P}$. (2) At the maximum $E_{P}$, the influences of the total number of TE elements and the total HEX area on the $E_{P}$ are analyzed, and the optimal distribution of HEXs area and optimal distribution of TE elements are found. (3) The optimal design scheme is obtained by comparing the deviation indices of different optimization objective combinations with the LINMAP, TOPSIS and Shannon entropy approaches. (4) The introduction of the $E_{P}$ objective provides a new candidate for the design optimization of TTEGs.

## 2. Model of TTEG

**Figure 1:**
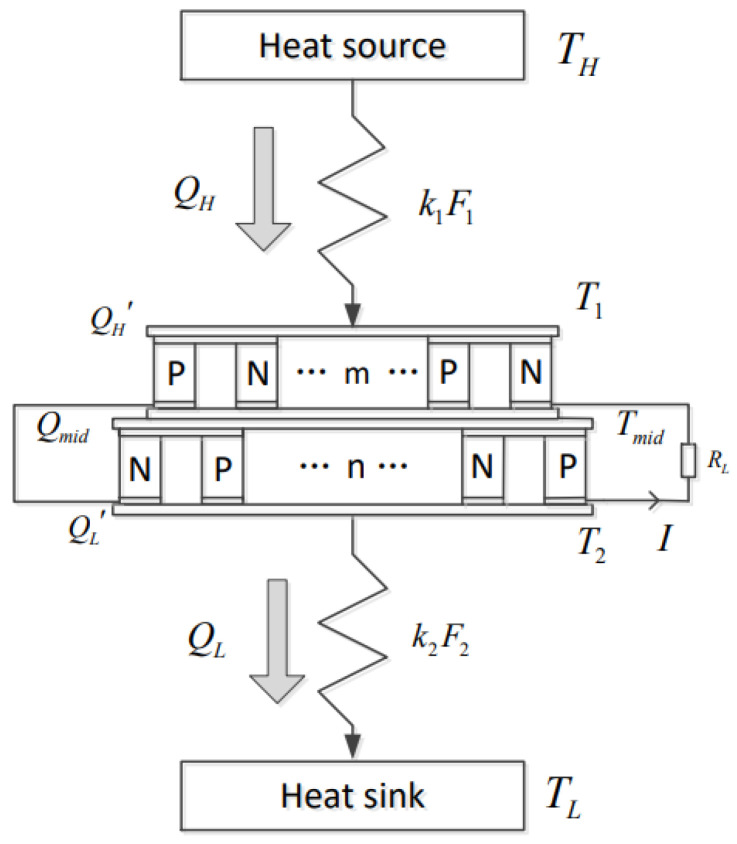
Model of TTEG [22].

Figure 1 is a model of the TTEG [22]. m and n are the number of TE elements in the first and second stages of the TTEG, respectively. M is the total number of TE elements. $T_{H}$ and $T_{L}$ are high- and low-temperature heat reservoir temperatures of the TTEG, respectively. The heat flow rate from high-temperature heat source to the TTEG is $Q_{H}$, and the heat flow rate from the TTEG to the low-temperature heat sink is $Q_{L}$. $T_{1}$, $T_{mid}$ and $T_{2}$ are the temperatures of the hot end of the first stage, the middle connecting layer and the second-stage cold end of the TTEG, respectively. ${Q^{\prime }}_{H}$, $Q_{mid}$ and ${Q^{\prime }}_{L}$ are the heat flow rates of the first-stage hot end, the middle connecting layer and the second-stage cold end of the TTEG, respectively. $R_{L}$ is the external load resistance of the TTEG, and I is the output current.

Considering the general structure of the TTEG and reducing the computational complexity, the following assumptions are made for the model:

(1) The effects of convective and radiative HT between and outside the TE element are ignored.
(2) The effect of the Thomson effect is ignored.
(3) The contact surface temperature difference and heat leakage losses between the first and second stages are ignored.
(4) When the temperature difference at all stages is not large, the performance of the TE elements at all stages does not change with temperature.

The heat flow rates of the first-stage hot end, the middle connecting layer and the second-stage cold end of the TTEG are, respectively

(1) $$Q_{H}{}^{\prime }=m(\alpha IT_{1}+K(T_{1}-T_{mid})-\frac{1}{2}I^{2}R)$$

(2) $$Q_{mid}=m(\alpha IT_{mid}+K(T_{1}-T_{mid})+\frac{1}{2}I^{2}R)$$

(3) $$Q_{mid}=n(\alpha IT_{mid}+K(T_{mid}-T_{2})-\frac{1}{2}I^{2}R)$$

(4) $$Q_{L}{}^{\prime }=n(\alpha IT_{2}+K(T_{mid}-T_{2})+\frac{1}{2}I^{2}R)$$

where α is the Seebeck coefficient of the TE elements, R and K are the thermal conductivity and electrical resistance of the TE elements.

The first law of thermodynamics gives

(5) $$Q_{H}=k_{1}F_{1}(T_{H}-T_{1})=Q_{H}{}^{\prime }$$

(6) $$Q_{L}=k_{2}F_{2}(T_{2}-T_{L})=Q_{L}{}^{\prime }$$

where $k_{1}$ and $k_{2}$ are the HT coefficients, and $F_{1}$ and $F_{2}$ are the HT areas of the HEXs on both sides.

According to Equations (2) and (3), the expression of $T_{mid}$ is

(7) $$T_{mid}=\frac{mKT_{1}+nKT_{2}+(m+n)I^{2}R/2}{K(m+n)+\alpha I(n-m)}$$

According to Equations (1), (4)–(7), the expressions of $T_{1}$, $T_{2}$, $Q_{1}$ and $Q_{2}$ are

(8) $$T_{1}=\frac{\begin{array}{c} \left\{\left[K(m+n)+\alpha I(n-m)\right](k_{1}F_{1}T_{H}+mI^{2}R/2)+(m+n)mKI^{2}R/2\right\}\\ \left(k_{2}F_{2}+nK-n\alpha I)+nK^{2}(mk_{2}F_{2}T_{L}-nk_{1}F_{1}T_{H}\right) \end{array}}{\begin{array}{c} \left[K(m+n)+\alpha I(n-m)\right](k_{1}F_{1}+mK+m\alpha I)(k_{2}F_{2}+nK-n\alpha I)\\ -{\left(nK\right)}^{2}(k_{1}F_{1}+mK+m\alpha I)-{\left(mK\right)}^{2}(k_{2}F_{2}+nK-n\alpha I) \end{array}}$$

(9) $$T_{2}=\frac{\begin{array}{c} \left\{\left[K(m+n)+\alpha I(n-m)\right](k_{2}F_{2}T_{L}+nI^{2}R/2)+(m+n)nKI^{2}R/2\right\}\\ \left(k_{1}F_{1}+mK+m\alpha I)-mK^{2}(mk_{2}F_{2}T_{L}-nk_{1}F_{1}T_{H}\right) \end{array}}{\begin{array}{c} \left[K(m+n)+\alpha I(n-m)\right](k_{1}F_{1}+mK+m\alpha I)(k_{2}F_{2}+nK-n\alpha I)\\ -{\left(nK\right)}^{2}(k_{1}F_{1}+mK+m\alpha I)-{\left(mK\right)}^{2}(k_{2}F_{2}+nK-n\alpha I) \end{array}}$$

(10) $$Q_{H}=\frac{mk_{1}F_{1}\left\{\begin{array}{c} \left[K(m+n)+\alpha I(n-m)\right](nK+k_{2}F_{2}-n\alpha I)(\alpha IT_{H}-I^{2}R/2+KT_{H})\\ -(m+n)(k_{2}F_{2}+nK-n\alpha I)KI^{2}R/2-{\left(nK\right)}^{2}(\alpha I+K)T_{H}\\ -K^{2}\left[k_{2}F_{2}(mT_{H}+nT_{L})+mn(K-\alpha I)T_{H}\right] \end{array}\right\}}{\begin{array}{c} \left[K(m+n)+\alpha I(n-m)\right](mK+k_{1}F_{1}+m\alpha I)(nK+k_{2}F_{2}-n\alpha I)\\ -{\left(nK\right)}^{2}(k_{1}F_{1}+m\alpha I+mK)-{\left(mK\right)}^{2}(k_{2}F_{2}+nK-n\alpha I) \end{array}}$$

(11) $$Q_{L}=\frac{nk_{2}F_{2}\left\{\begin{array}{c} \left[K(m+n)+\alpha I(n-m)\right](mK+k_{1}F_{1}+m\alpha I)(\alpha IT_{L}+I^{2}R/2-KT_{L})\\ +(m+n)(mK+k_{1}F_{1}+m\alpha I)KI^{2}R/2+{\left(mK\right)}^{2}(K-\alpha I)T_{L}\\ +K^{2}\left[k_{1}F_{1}(mT_{H}+nT_{L})+mn(K+\alpha I)T_{L}\right] \end{array}\right\}}{\begin{array}{c} \left[K(m+n)+\alpha I(n-m)\right](mK+k_{1}F_{1}+m\alpha I)(nK+k_{2}F_{2}-n\alpha I)\\ -{\left(nK\right)}^{2}(k_{1}F_{1}+m\alpha I+mK)-{\left(mK\right)}^{2}(k_{2}F_{2}+nK-n\alpha I) \end{array}}$$

Equations (8) and (9) reflect the relationships among the temperatures of the hot and cold sides as well as the number of TE elements, internal losses, external heat reservoir temperatures and the external HT irreversibility. It can be seen that when the design parameters change, the temperatures of the hot and cold surfaces will also change. Therefore, the temperatures of the hot and cold sides are not constants.

According to Equations (1)–(11), one has

(12) $$B_{4}I^{4}+B_{3}I^{3}+B_{2}I^{2}+B_{1}I+B_{0}=0$$

where $B_{4}$, $B_{3}$, $B_{2}$, $B_{1}$ and $B_{0}$ are coefficients shown in Appendix A. According to Equation (12), the stable current I of the device can be obtained. From Equations (A1)–(A5), it can be known that coefficients $B_{4}$, $B_{3}$, $B_{2}$, $B_{1}$ and $B_{0}$ are related to the number of TE elements and physical parameters (n, m, α, K and R), heat reservoir temperatures ($T_{H}$ and $T_{L}$) and the HEX parameters ($k_{1}$, $F_{1}$, $k_{2}$ and $F_{2}$), so I is a function of the physical parameters of each TE element.

According to Reference [22], the expressions of P and η are

(13) $$P=Q_{H}-Q_{L}$$

(14) $$\eta =\frac{P}{Q_{H}}=1-\frac{Q_{L}}{Q_{H}}$$

According to the References [26,27,28], the $E_{P}$ is defined as

(15) $$E_{P}=P\eta =\frac{{\left(Q_{H}-Q_{L}\right)}^{2}}{Q_{H}}$$

(16) $$E_{P}=\frac{{\left\{\begin{array}{c} \left[K(m+n)+\alpha I(n-m)\right](mk_{1}F_{1}k_{2}F_{2}+mnKk_{1}F_{1}-mnk_{1}F_{1}\alpha I)(KT_{H}+\alpha IT_{H}-I^{2}R/2)\\ -KI^{2}R/2(m+n)\left[\left(m+n)k_{1}F_{1}k_{2}F_{2}+mnK(k_{1}F_{1}+k_{2}F_{2})+mn\alpha I(k_{1}F_{1}-k_{2}F_{2}\right)\right]\\ -(nk_{1}F_{1}k_{2}F_{2}+mnKk_{2}F_{2}+mnk_{2}F_{2}\alpha I)(\alpha IT_{L}-KT_{L}+I^{2}R/2)\\ -mnK^{3}(m+n)(k_{1}F_{1}T_{H}+k_{2}F_{2}T_{L})-(m+n)k_{1}F_{1}k_{2}F_{2}(mT_{H}+nT_{L})\\ +mn\alpha IK^{2}(m-n)(k_{1}F_{1}T_{H}+k_{2}F_{2}T_{L}) \end{array}\right\}}^{2}}{\begin{array}{c} mk_{1}F_{1}\left\{\begin{array}{c} \left[K(m+n)+\alpha I(n-m)\right](nK+k_{2}F_{2}-n\alpha I)(\alpha IT_{H}-I^{2}R/2+KT_{H})\\ -(m+n)(k_{2}F_{2}+nK-n\alpha I)KI^{2}R/2-{\left(nK\right)}^{2}(\alpha I+K)T_{H}\\ -K^{2}\left[k_{2}F_{2}(mT_{H}+nT_{L})+mn(K-\alpha I)T_{H}\right] \end{array}\right\}\\ \left\{\begin{array}{c} \left[K(m+n)+\alpha I(n-m)\right](mK+k_{1}F_{1}+m\alpha I)(nK+k_{2}F_{2}-n\alpha I)\\ -{\left(nK\right)}^{2}(k_{1}F_{1}+m\alpha I+mK)-{\left(mK\right)}^{2}(k_{2}F_{2}+nK-n\alpha I) \end{array}\right\} \end{array}}$$

It can be seen from Equation (15) that the $E_{P}$ is related to external parameters such as m, n, $k_{1}$, $k_{2}$, $F_{1}$, $F_{2}$, $T_{H}$ and $T_{L}$. This paper mainly studies the distribution of the TE elements and the effect of external HT losses. The internal parameters such as the height of the TE legs do have a great influence on the performance of the TTEG, refer to References [45,46,47,48,49,50,51,52,53]. If more parameters are considered, the results will be more in line with the actual operation of the TTEG. The authors will consider the effect of more internal parameters on TTEG performance in future studies.

## 3. Efficient Power Performance Analysis

### 3.1. Optimal Distribution of Heat Exchangers Area 

Assuming that the sum ($F_{T}$) of the area of the two HEXs is constant

(17) $$F_{T}=F_{1}+F_{2}$$

The distribution (f) of HEXs area is defined as

(18) $$f=F_{1}/F_{T}$$

Determining the initial value according to References [22,54]: $T_{H}=600K$, $T_{L}=300K$, $\alpha =2.3\times 10^{-4}V/K$, $F_{T}=0.07m^{2}$, $R=1.4\times 10^{-3}\Omega$, $K=1.5\times 10^{-2}W/K$, M=80, m=39, n=41, $k_{1}=60W/(m^{2}\cdot K)$, $k_{2}=15W/(m^{2}\cdot K)$ and $F_{T}=0.07m^{2}$.

**Figure 2:**
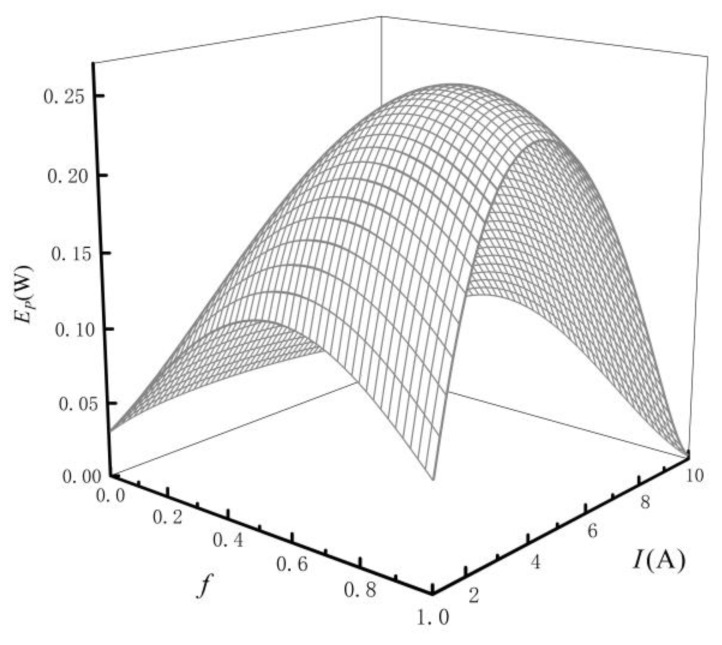
Relations of $E_{P}$ versus f and I.

**Figure 3:**
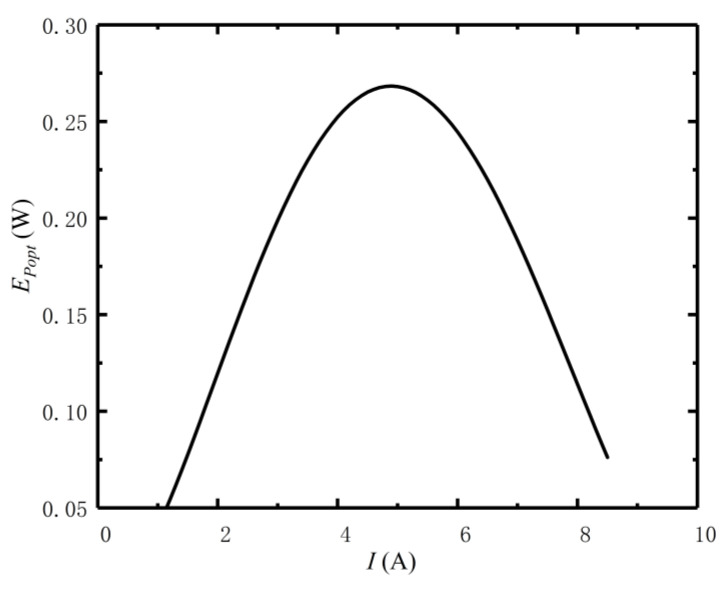
Relations of $E_{Popt}$ versus I under condition $f_{opt}$.

Figure 2 reflects the relationships of $E_{P}$ versus f and I. From Figure 2, when I is constant, the $E_{P}$ and f are parabolic-like, and there is an optimal distribution ($f_{opt}$) of HEX area to achieve the optimal $E_{P}$ ($E_{Popt}$). When f is fixed, the $E_{P}$ and I are parabolic-like, and there is an optimal current ($I_{opt}$) to achieve $E_{Popt}$. Figure 3 reflects the relationship of $E_{Popt}$ versus I under condition of $f_{opt}$. From Figure 3, there is an optimum current to achieve a maximum for the $E_{P}$ ($E_{P\max}$), the peak is shown in Figure 2.

**Figure 4:**
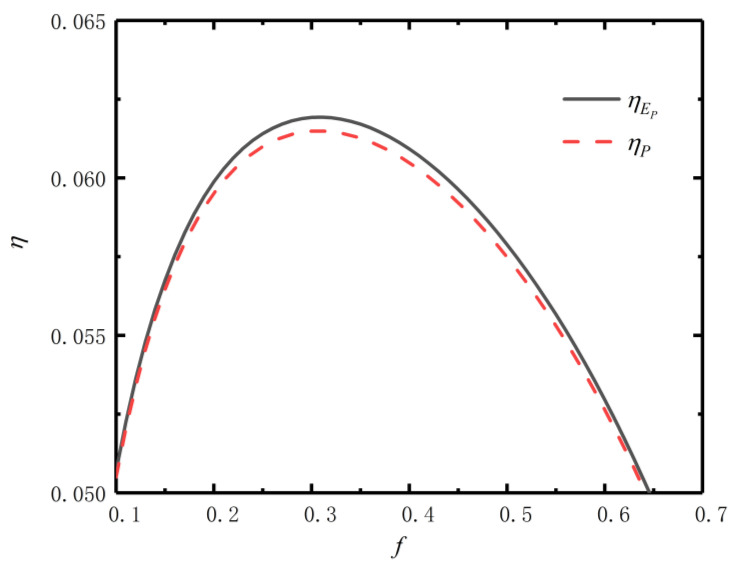
Relations of f versus $\eta_{P}$ and $\eta_{E_{P}}$.

Figure 4 reflects the relationships of f versus efficiency ($\eta_{P}$) at maximum P ($P_{\max}$) and efficiency ($\eta_{E_{P}}$) at maximum $E_{P}$ ($E_{P\max}$). From Figure 4, the efficiency ($\eta_{E_{P}}$) corresponding to the $E_{P\max}$ is significantly higher than the efficiency ($\eta_{P}$) corresponding to the $P_{\max}$. Therefore, taking the $E_{P}$ as the optimization objective, the η of the TTEG can be improved.

### 3.2. Effect of Number and Distribution of Thermoelectric Elements on Efficient Power 

The total number (M) of TE elements is limited

(19) M=m+n

The distribution (x) of TE elements is defined as

(20) $$x=\frac{m}{M}$$

The first- and second-stage TE element numbers (m and n) in a TTEG must be positive integers. Therefore, the smallest step size of the variation of m and n is 1, and x is not a continuous number.

Table 1 lists the optimum distribution of thermoelectric elements for $E_{P\max}$. Under different total TE element numbers, the number of first-stage TE elements of the TTEG is always smaller than that of the second-stage TE element numbers when the efficient power reaches the maximum. As M increases, x increases, and x gets closer and closer to 0.5. Therefore, when designing a TTEG, so as to improve the performance of the TTEG, the number of TE elements in the first stage should be smaller than that in the second stage. 

**Figure 5:**
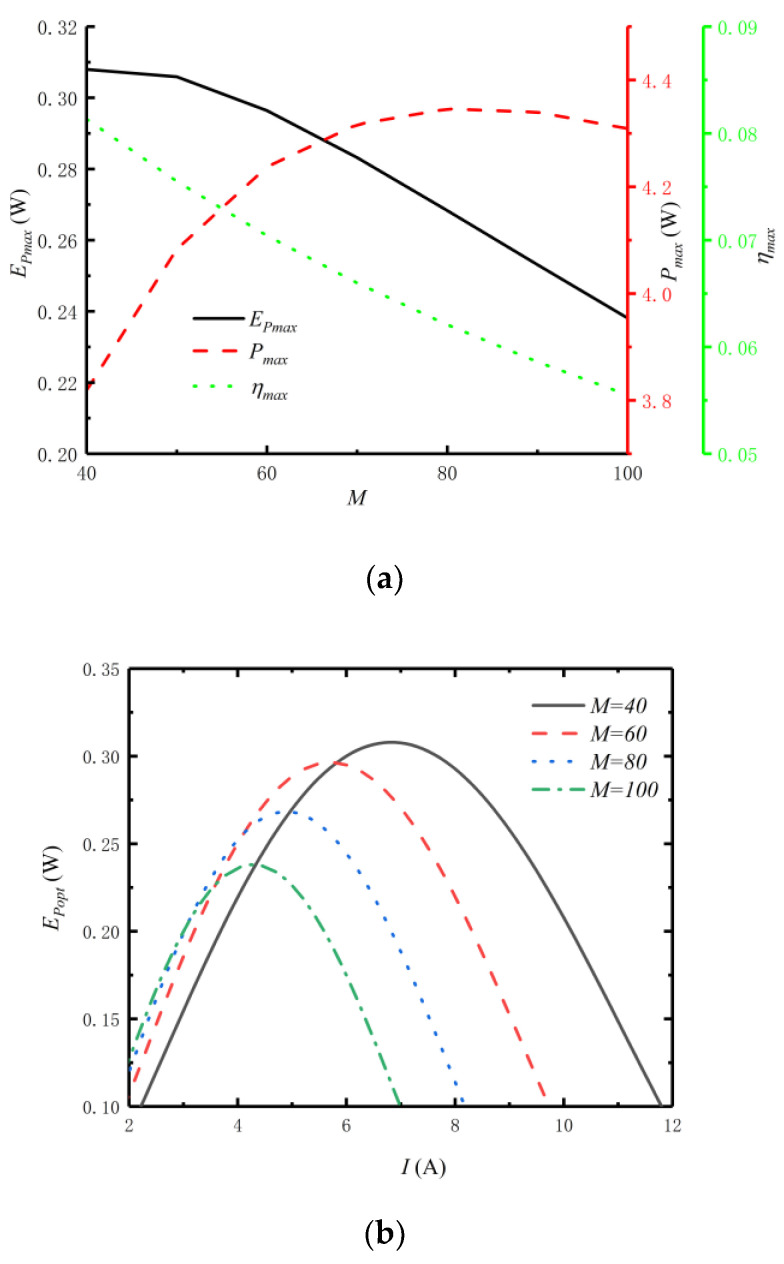

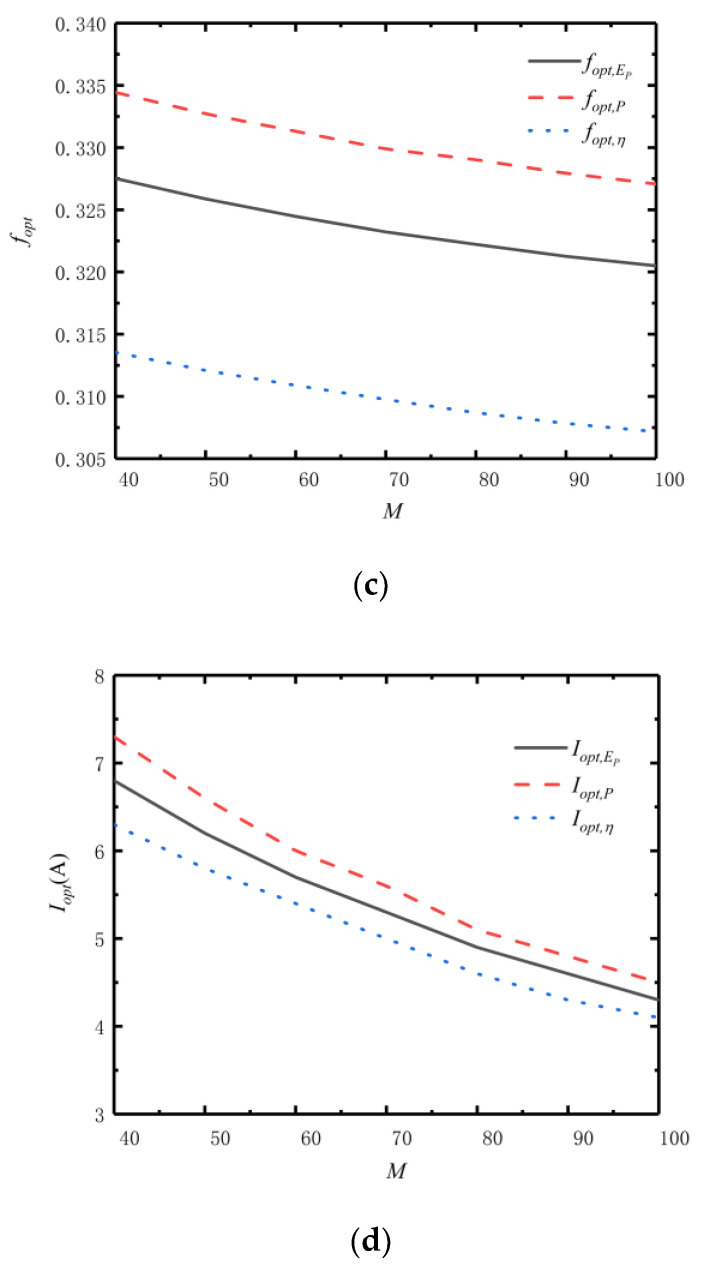
Relations of $P_{\max}$, $\eta_{\max}$, $E_{P\max}$, $f_{opt}$ and $I_{opt}$ versus M. (**a**) Relations of $P_{\max}$, $\eta_{\max}$ and $E_{P\max}$ versus M. (**b**) $E_{P\max}$ versus I under different M. (**c**) Relations of $f_{opt}$ versus M. (**d**) Relations of $I_{opt}$ versus M.

Figure 5 reflects the relationships of $P_{\max}$, $\eta_{\max}$, $E_{P\max}$, $f_{opt}$ and $I_{opt}$ versus M. Figure 5a reflects the relationships among $P_{\max}$, $\eta_{\max}$ and $E_{P\max}$ and M. From Figure 5a, as M increases, the $P_{\max}$ first increases and then decreases, the $E_{P\max}$ increases, and the $\eta_{\max}$ decreases. Therefore, in the actual design of a TTEG, it is not necessary to increase M to improve the performance of the TTEG, but to select the appropriate M by taking into account P, η and $E_{P}$ at the same time. Figure 5b reflects the effect of M on $E_{Popt}-I$ at optimal distribution of HEX area. From Figure 5b, the $E_{P\max}$ increases as the M increases. When the M increases from 40 to 100, the $E_{P\max}$ decreases from 0.308W to 0.2381W, a decrease of 22.7%. The corresponding $I_{opt}$ is reduced from 6.8A to 4.3A, which is reduced by 36.8%. Therefore, when the total number of TE elements changes, it has a greater impact on the efficient power and optimal current.

Figure 5c,d reflect relationships of $f_{opt}$ and $I_{opt}$ versus M. From Figure 5c, as M increases, the optimal distributions ($f_{opt,P}$, $f_{opt,\eta }$ and $f_{opt,E_{P}}$) of HEX area for output power, thermal efficiency and efficient power all decrease, and $f_{opt,P}>f_{opt,E_{P}}>f_{opt,\eta }$. When M increases from 40 to 100, $f_{opt,P}$ decreases from 0.3344 to 0.3271, a decrease of 2.2%, $f_{opt,\eta }$ decreases from 0.3136 to 0.3072, a decrease of 2.0%, and $f_{opt,E_{P}}$ decreases from 0.3275 to 0.3205, a decrease of 2.1%. From Figure 5d, as M increases, the optimal currents ($I_{opt,P}$, $I_{opt,\eta }$ and $I_{opt,E_{P}}$) for output power, thermal efficiency and efficient power all decrease, and $I_{opt,P}>I_{opt,E_{P}}>I_{opt,\eta }$. When M increases from 40 to 100, $I_{opt,P}$ decreases from 7.3A to 4.5A, a decrease of 38.4%, $I_{opt,\eta }$ decreases from 6.3A to 4.1A, a decrease of 34.9%, and $I_{opt,E_{P}}$ decreases from 6.8A to 4.3A, a decrease of 36.8%.

### 3.3. Effect of Total Heat Exchanger Area on Efficient Power

**Figure 6:**
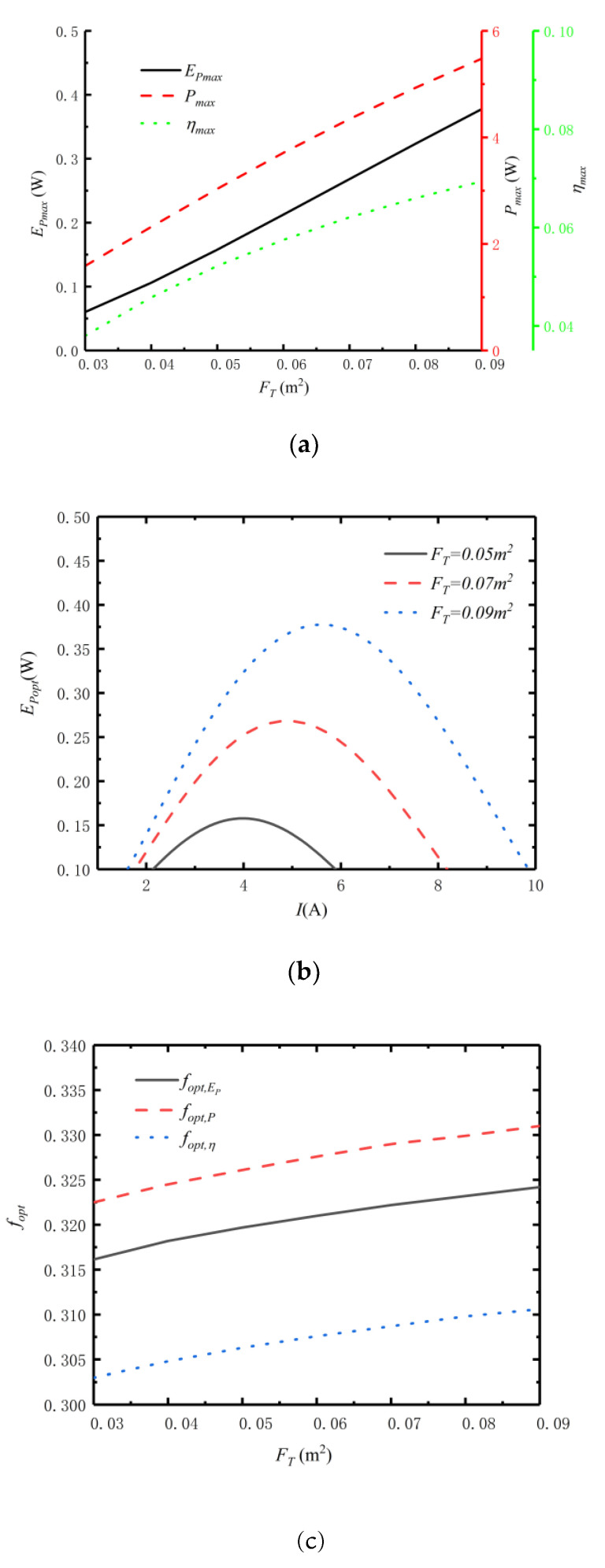

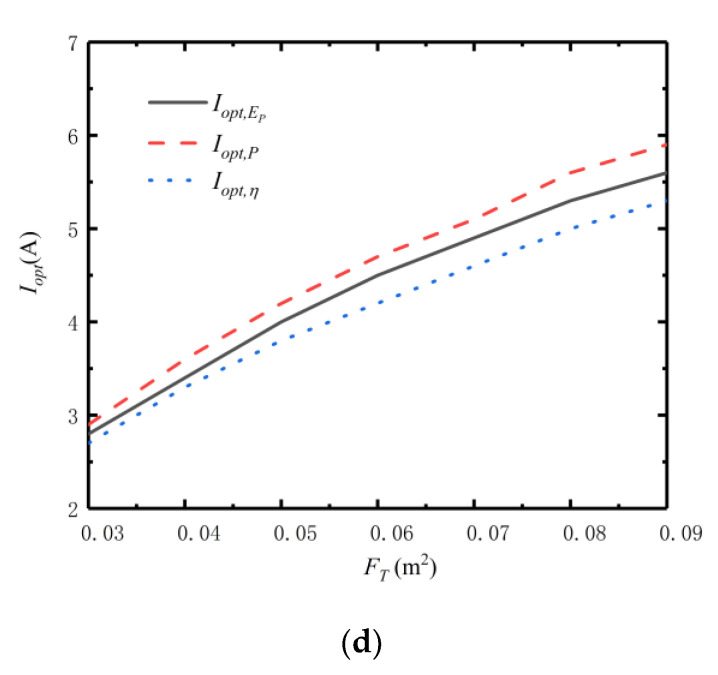
Relations of $P_{\max}$, $\eta_{\max}$, $E_{P\max}$, $f_{opt}$ and $I_{opt}$ versus $F_{T}$. (**a**) Relations of $P_{\max}$, $\eta_{\max}$ and $E_{P\max}$ versus $F_{T}$. (**b**) $E_{P\max}$ versus I under different $F_{T}$. (**c**) Relations of $f_{opt}$ versus $F_{T}$. (**d**) Relations of $I_{opt}$ versus $F_{T}$.

Figure 6 reflects the relationships of $P_{\max}$, $\eta_{\max}$, $E_{P\max}$, $f_{opt}$ and $I_{opt}$ versus total HEX area ($F_{T}$). Figure 6a reflects the relationships among $P_{\max}$, $\eta_{\max}$ and $E_{P\max}$ and $F_{T}$. From Figure 6a, as $F_{T}$ increases, $P_{\max}$, $\eta_{\max}$ and $E_{P\max}$ all increase. Therefore, in the actual design of the TTEG, the total HEX area should be increased as much as possible when the size of the TTEG is constant. Figure 6b reflects the effect of $F_{T}$ on $E_{P\max}-I$ at optimal distribution of HEXs area. From Figure 6b, the $E_{P\max}$ increases with the increase in the $F_{T}$. When the $F_{T}$ increases from $0.05m^{2}$ to $0.09m^{2}$, the $E_{P\max}$ increases from 0.1578W to 0.3777W, an increase of 139.4%. The corresponding $I_{opt}$ increases from 4.0A to 5.6A, which is increased by 40%. Therefore, when the total HEX area changes, it has a greater impact on the efficient power and optimal current.

Figure 6c,d reflect relationships of $f_{opt}$ and $I_{opt}$ versus $F_{T}$. From Figure 6c, as $F_{T}$ increases, the optimal distributions ($f_{opt,P}$, $f_{opt,\eta }$ and $f_{opt,E_{P}}$) of HEX area for output power, thermal efficiency and efficient power all increase, and $f_{opt,P}>f_{opt,E_{P}}>f_{opt,\eta }$. When $F_{T}$ increases from $0.05m^{2}$ to $0.09m^{2}$, $f_{opt,P}$ increases from 0.3261 to 0.331, an increase of 1.5%, $f_{opt,\eta }$ increases from 0.3063 to 0.3106, an increase of 1.4%, and $f_{opt,E_{P}}$ increases from 0.3197 to 0.3242, an increase of 1.4%. From Figure 6d, as $F_{T}$ increases, the optimal currents ($I_{opt,P}$, $I_{opt,\eta }$ and $I_{opt,E_{P}}$) for output power, thermal efficiency and efficient power all increase, and $I_{opt,P}>I_{opt,E_{P}}>I_{opt,\eta }$. When $F_{T}$ increases from $0.05m^{2}$ to $0.09m^{2}$, $I_{opt,P}$ increases from 4.2A to 5.9A, an increase of 40.5%; $I_{opt,\eta }$ increases from 3.8A to 5.3A, an increase of 39.5%; and $I_{opt,E_{P}}$ increases from 4.0A to 5.6A, an increase of 40%. 

## 4. Multi-Objective Optimization

**Figure 7:**
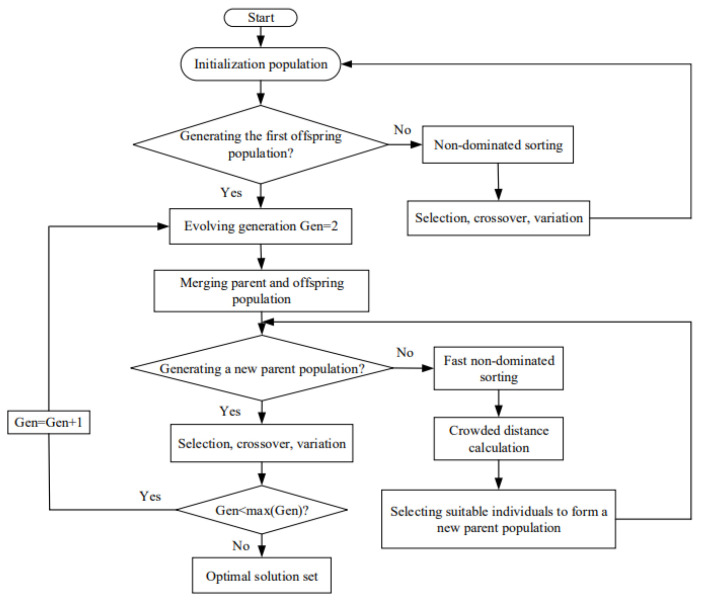
Flowchart of NSGA-II algorithm.

It can be seen from Figure 5c,d and Figure 6c,d that the optimal current and the optimal distribution of the HEX area are all different at $P_{\max}$, $\eta_{\max}$ and $E_{P\max}$, so it is impossible to simultaneously achieve the $P_{\max}$, $\eta_{\max}$ and $E_{P\max}$ under the same current and area distribution. When designing a TTEG, obtaining the maximum performance values with the minimum total HEX area is the optimum design. This is approached by MOO with the optimal distribution of the HEX area. MOO does not mean that each optimization objective reaches the maximum value. The basic idea of the non-dominated sorting genetic algorithm II (NSGA-II) [55] is as follows: First, an initial population of size N is randomly generated. After non-dominated sorting, the first generation population is obtained through the three basic operations of genetic algorithm, selection, crossover and mutation. Second, starting from the second generation population, the parent population and the child population are merged to perform a fast non-dominated sorting, and the crowding degree of each individual in the non-dominated layer is calculated. Individuals form a new generation of parent populations. Finally, a new generation of progeny population is generated by the genetic algorithm, and the optimization process is carried out in this form until the end condition is met. The corresponding flowchart is shown in Figure 7. Its essence is to balance the advantages and disadvantages of each optimization objective through NSGA-II to achieve the best compromise of different optimization objectives and obtain a series of feasible solutions. It is also called the Pareto frontier. After obtaining the results of different optimization objective combinations, the deviation index (DI) is compared for three decision methods, LINMAP [56], TOPSIS [57,58] and Shannon entropy [59]. Based on the results of the NSGA-II algorithm, the DI [60] is used to characterize the closeness between the optimal solution and the positive ideal point. The smaller the DI, the closer to the positive ideal point. The positive ideal point is the point where the corresponding P, η and $E_{P}$ are all at maximum.

Determining the initial value according to References [22,54]: $T_{H}=600K$, $T_{L}=300K$, $\alpha =2.3\times 10^{-4}V/K$, $F_{T}=0.07m^{2}$, $R=1.4\times 10^{-3}\Omega$, $K=1.5\times 10^{-2}W/K$, M=80, m=39, n=41, $k_{1}=60W/(m^{2}\cdot K)$, $k_{2}=15W/(m^{2}\cdot K)$ and $F_{T}=0.07m^{2}$. In this paper, for the convenience of data processing, the method of Reference [61] is used to perform dimensionless processing on the output power and efficient power to obtain the dimensionless output power ($\bar{P}$) and dimensionless efficient power (${\bar{E}}_{P}$).

(21) $$\bar{P}=P/P_{\max}$$

(22) $${\bar{E}}_{P}=E_{P}/E_{P\max}$$

This paper takes I, f and x as optimization variables, and $\bar{P}$, η and ${\bar{E}}_{P}$ as optimization objectives, to perform MOO for the TTEG. The value ranges of the optimization variables are

(23) 0<I<10A

(24) 0<f<1

(25) $$x\in \{\frac{1}{M},\frac{2}{M},\frac{3}{M},\cdots ,\frac{M-1}{M}\}$$

Table 2 lists the comparison of the optimal solutions obtained by MOOs and single-objective optimization. From Table 2, the DIs are 0.1866, 0.1866 and 0.1815 with the LINMAP, TOPSIS and Shannon entropy approaches, respectively, when the MOO is performed with three-objective optimization ($\bar{P}-\eta -{\bar{E}}_{P}$). The DIs are 0.2140, 0.9429 and 0.1815, respectively, for three single-objective optimizations of maximum $\bar{P}$, η and ${\bar{E}}_{P}$. The DI of the three decision-making methods in the three-objective optimization is smaller than the DIs of $\bar{P}$ and η single-objective optimizations. The three-objective optimization Shannon entropy decision-making method has the same DI as the single-objective $\bar{P}$ optimization, and the results are equally good. It indicates that compared with single-objective optimization, MOO can better take into account different optimization objectives by choosing appropriate decision-making methods.

**Figure 8:**
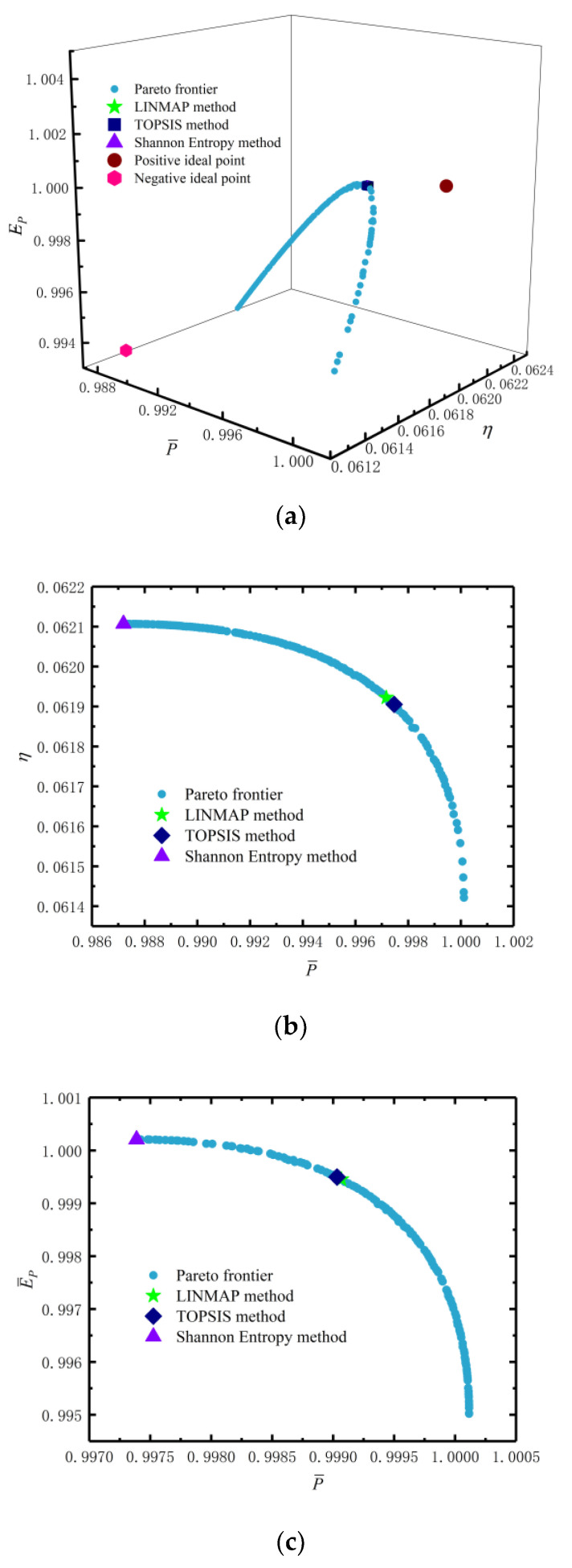

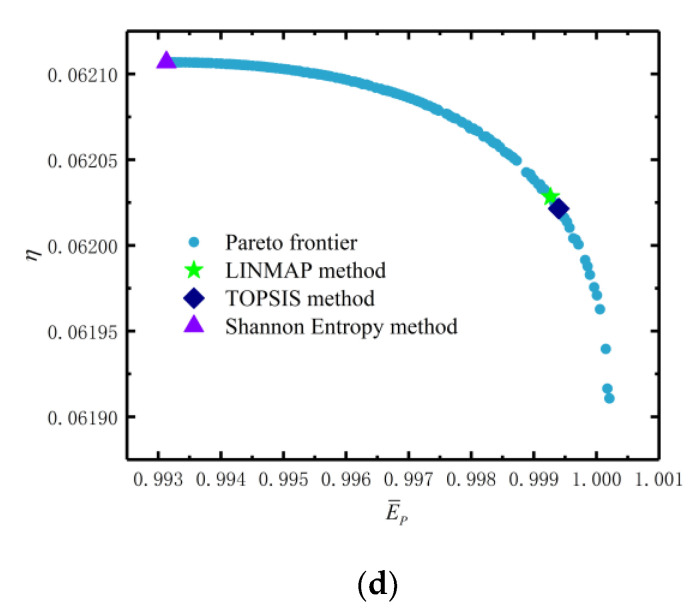
Results of three-objective and two-objective optimizations. (**a**) Pareto frontier of $\bar{P}-\eta -{\bar{E}}_{P}$. (**b**) Pareto frontier of $\bar{P}-\eta$. (**c**) Pareto frontier of $\bar{P}-{\bar{E}}_{P}$. (**d**) Pareto frontier of $\eta -{\bar{E}}_{P}$.

Figure 8 reflects the corresponding Pareto frontiers when multiple objectives are simultaneously optimized. Figure 8a reflects the Pareto frontier corresponding to the three-objective ($\bar{P}-\eta -{\bar{E}}_{P}$) optimization. The brown-red circles represent the positive ideal point, and the pink hexagons represent the negative ideal point. Green pentagonal blue squares and purple triangles represent the corresponding points of the LINMAP, TOPSIS and Shannon entropy decision schemes, respectively. From Figure 8a, the corresponding points of the three decision-making methods almost coincide. As $\bar{P}$ increases, η gradually decreases, and ${\bar{E}}_{P}$ increases first and then decreases. Figure 8b–d reflect the Pareto frontiers corresponding to three two-objective ($\bar{P}-\eta$, $\bar{P}-{\bar{E}}_{P}$ and $\eta -{\bar{E}}_{P}$) optimizations. It can be seen that $\bar{P}$ decreases with the increases in η and ${\bar{E}}_{P}$, and as η increases, ${\bar{E}}_{P}$ decreases. 

**Figure 9:**
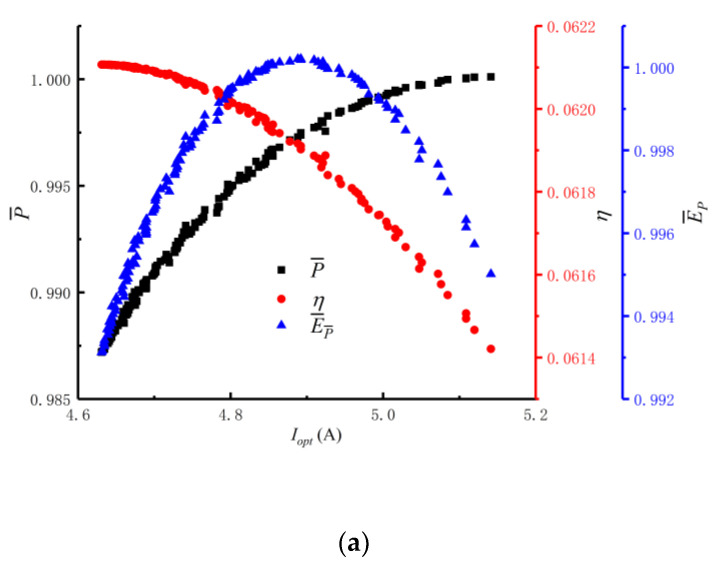

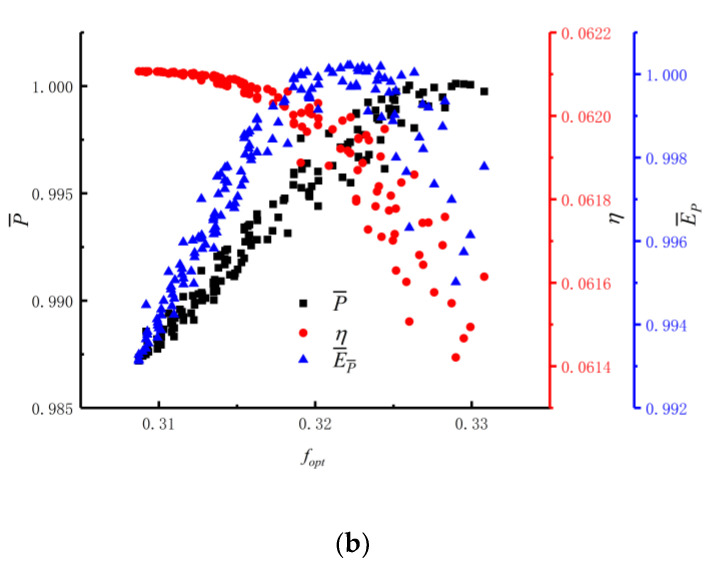
$I_{opt}$ and $f_{opt}$ distributions corresponding to the Pareto frontiers. (**a**) Distribution of $I_{opt}$. (**b**) Distribution of $f_{opt}$.

For the MOO of $\bar{P}-\eta -{\bar{E}}_{P}$, the distributions ($x_{opt}$) are 38/80 and 39/80, and most values are 39/80. The change trend cannot be represented in the distribution figure. Therefore, the distributions of $x_{opt}$ are not plotted in Figure 9. Figure 9 reflects the distributions of $I_{opt}$ and $f_{opt}$ corresponding to the Pareto frontier during optimizations. Figure 9a reflects that $I_{opt}$ is distributed between 4.6A and 5.2A, as $I_{opt}$ increases, $\bar{P}$ increases, η decreases, ${\bar{E}}_{P}$ first increases and then decreases. Figure 9b reflects that $f_{opt}$ is distributed between 0.305 and 0.335. The corresponding points of $\bar{P}$, η and ${\bar{E}}_{P}$ are scattered. With the increase in $f_{opt}$, the general trend of $\bar{P}$ is increasing, the general trend of η is decreasing and the general trend of ${\bar{E}}_{P}$ is to increase first and then decrease. 

**Figure 10:**
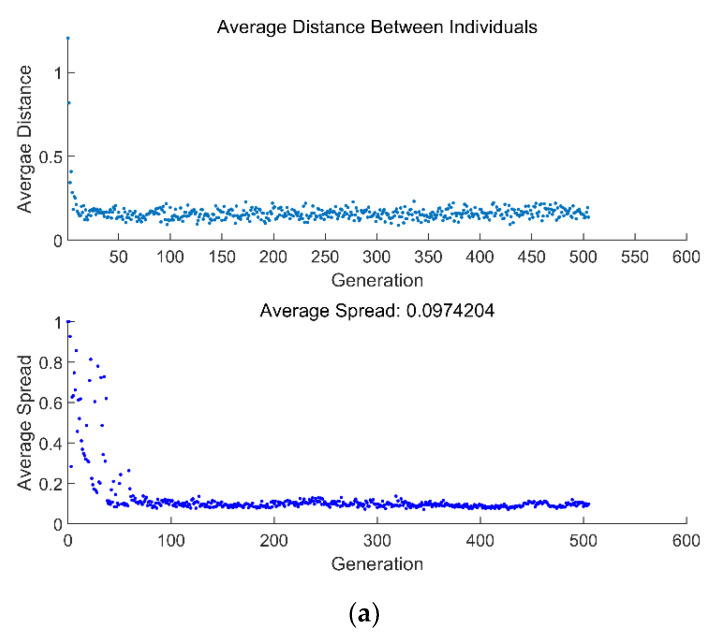

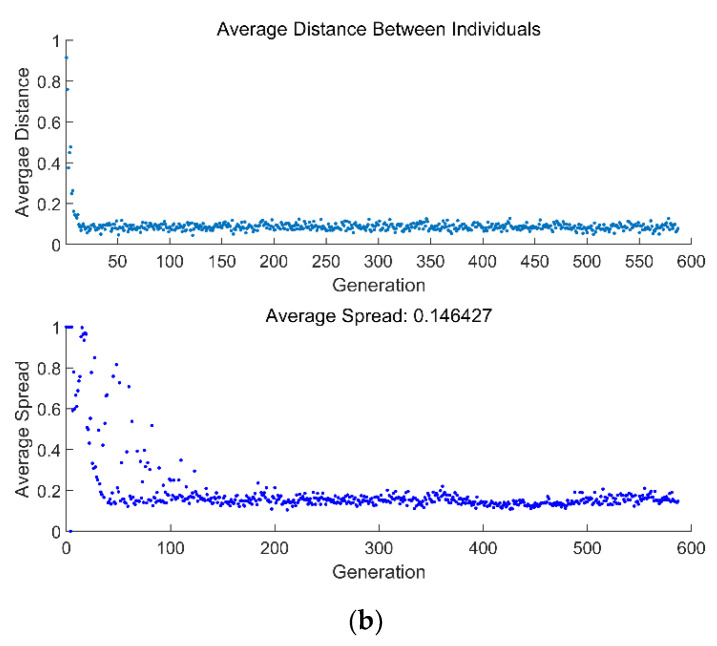
Average distance and average spread versus number of generations. (**a**) $\bar{P}-\eta -{\bar{E}}_{P}$. (**b**) $\bar{P}-\eta$.

Figure 10 reflects the average spread and generation number of three-objective $\bar{P}-\eta -{\bar{E}}_{P}$ and two-objective $\bar{P}-\eta$. From Figure 10a, the three-objective ($\bar{P}-\eta -{\bar{E}}_{P}$) optimization converges at the 511th generation, and the genetic algorithm stops. From Figure 10b, the two-objective ($\bar{P}-\eta$) optimization converges at the 583rd generation, and the genetic algorithm stops.

## 5. Conclusions

Based on the TTEG model established in Reference [22], this paper deduces the $E_{P}$ expression of the TTEG. For a fixed total number of TE elements and a fixed total HEX area, the optimal distributions of TE elements and HEX area are optimized for $E_{P}$. The NSGA-II algorithm is used to optimize $\bar{P}$, η and ${\bar{E}}_{P}$ with MOO, and the Pareto frontier with the optimal solution set is obtained. The main conclusions are:

1. The efficiency at maximum efficient power is significantly higher than the efficiency at maximum power. Under the same distribution of HEXs area, the efficiency at maximum efficient power and efficiency at maximum power both reach the maximum. The efficient power takes into account both the output power and thermal efficiency, and can provide new ideas for the design of practical TTEGs.
2. It is found that reducing the total number of TE elements and increasing the total HEX area can not only obtain a higher output current, but also higher efficient power.
3. By comparing the DIs of three-objective optimization and the DIs of single-objective optimization, it can be found that the former has a lower DI. Therefore, taking the three-objective optimization as the design scheme, the performance of the TTEG will be optimized.
4. It is also found that in the MOO, there are optimal intervals for the distribution of the TE elements, output current and distribution of HEX area. Values in these ranges will significantly improve the various performances of the TTEGs.

## Figures and Tables

**Table 1:** Optimum distribution of thermoelectric elements.

M	m	n	$E_{P\max}(W)$	$x_{opt}$
40	19	21	0.308W	0.475
50	24	26	0.3059W	0.48
60	29	31	0.2964W	0.4833
70	34	36	0.2832W	0.4857
80	39	41	0.2683W	0.4875
90	44	46	0.2531W	0.4889
100	49	51	0.2381W	0.49

**Table 2:** Results of single-, two- and three-objective optimizations.

Optimization Objectives	Decision-Making Approach	Optimization Variables			Performance Indicators			Deviation Index
		I	f	x	$\bar{P}$	η	${\bar{E}}_{P}$	D
$\bar{P}$, η and ${\bar{E}}_{P}$	LINMAP	4.9	0.3221	39/80	0.9973	0.0619	1.0000	0.1866
	TOPSIS	4.9	0.3221	39/80	0.9973	0.0619	1.0000	0.1866
	Shannon entropy	4.9	0.3222	39/80	0.9974	0.0619	1.0000	0.1815
$\bar{P}$ and η	LINMAP	4.9	0.3221	39/80	0.9972	0.0619	1.0000	0.1943
	TOPSIS	4.9	0.3217	39/80	0.9975	0.0619	1.0000	0.1751
	Shannon entropy	4.6	0.3087	38/80	0.9872	0.0621	0.9937	0.9421
$\bar{P}$ and ${\bar{E}}_{P}$	LINMAP	5.0	0.3257	39/80	0.9991	0.0617	0.9995	0.0757
	TOPSIS	5.0	0.3247	39/80	0.9990	0.0617	0.9995	0.0934
	Shannon entropy	5.0	0.3223	39/80	0.9974	0.0618	1.0000	0.1821
η and ${\bar{E}}_{P}$	LINMAP	4.8	0.3183	39/80	0.9951	0.0620	0.9993	0.3366
	TOPSIS	4.8	0.3185	39/80	0.9951	0.0620	0.9994	0.3345
	Shannon entropy	4.6	0.3087	38/80	0.9872	0.0621	0.9931	0.9548
${\bar{P}}_{\max}$	——	5.1	0.3290	39/80	1.0000	0.0615	0.9966	0.2140
$\eta_{\max}$	——	4.6	0.3087	39/80	0.9872	0.0621	0.9925	0.9429
${\bar{E}}_{P\max}$	——	4.9	0.3222	39/80	0.9974	0.0619	1.0000	0.1815
Positive ideal point		——			1.0000	0.0621	1.0000	——
Negative ideal point		——			0.9872	0.0614	0.9931	——

## Nomenclature

$E_{P}$	Efficient power (*W*)
F	Heat transfer area of heat exchangers ($m^{2}$)
I	Output current (A)
K	Thermal conductance (W/K)
k	Coefficient of heat transfer ($W/m^{2}\cdot K$)
M	Total number of thermoelectric elements
m	Number of first-stage thermoelectric elements
n	Number of second-stage thermoelectric elements
P	Output power (W)
Q	Heat flux (W)
R	Electric resistance (Ω)
T	Temperature (K)
**Greek symbols**	
α	Total Seebeck coefficient (V/K)
η	Thermal efficiency
**Subscripts**	
1	High temperature side of thermoelectric device
2	Low temperature side of thermoelectric device
$E_{P}$	Maximum efficient power point
H	High-temperature heat source
L	Low-temperature heat sink
max	Maximum value
mid	Middle connection layer
opt	Optimal
P	Maximum power point
T	Total
η	Maximum thermal efficiency point
Superscript	
−	Dimensionless
**Abbreviations**	
DI	Deviation index
FTT	Finite-time thermodynamics
HEX	Heat exchanger
HT	Heat transfer
MOO	Multi-objective optimization
NET	Non-equilibrium thermodynamics
TE	Thermoelectric
TTEG	Two-stage thermoelectric generator

## Appendix A. Coefficients in Equation (12)

(A1)
$$B_{4}=0.5RmnT_{H}\alpha^{2}(n-m)$$

(A2)
$$B_{3}=RKmn\alpha T_{H}(m+n)-0.5Rm\alpha T_{H}(n-m)(k_{2}F_{2}+nK)-mnT_{H}\alpha^{3}(n-m)$$

(A3)
$$\begin{array}{cc} B_{2} & =mT_{H}\alpha^{2}(n-m)(nK+k_{2}F_{2})+nk_{1}F_{1}T_{H}\alpha^{2}(n-m)(T_{H}-1)\\ & \;-2mKT_{H}\alpha^{2}n^{2}-RKmT_{H}(m+n)(nK+k_{2}F_{2}) \end{array}$$

(A4)
$$\begin{array}{cc} B_{1} & =2mn\alpha T_{H}K(nK+k_{2}F_{2})+Kk_{1}F_{1}T_{H}\alpha n(m+n)(T_{H}-1)\\ & \;-2m\alpha T_{H}n^{2}K^{2}-k_{1}F_{1}T_{H}\alpha (n-m)(nK+k_{2}F_{2})(T_{H}-1) \end{array}$$

(A5)
$$\begin{array}{cc} B_{0} & =mT_{H}K^{2}(m+n)(nK+k_{2}F_{2})+Kk_{1}F_{1}k_{2}F_{2}T_{H}(m+n)(T_{H}-1)\\ & \;-mk_{2}F_{2}K^{2}(mT_{H}+nT_{L})-mnT_{H}K^{3}(m+n)\\ & \;-mnK^{2}(k_{1}F_{1}T_{H}+k_{2}F_{2}T_{L})(T_{H}-1) \end{array}$$
